# Phylogenetic, Functional and Safety Features of 1950s *B. infantis* Strains

**DOI:** 10.3390/microorganisms10020203

**Published:** 2022-01-18

**Authors:** Stéphane Duboux, Catherine Ngom-Bru, Florac De Bruyn, Biljana Bogicevic

**Affiliations:** 1 Société des Produits Nestlé SA, Nestlé Research, Route du Jorat 57, CH-1000 Lausanne 26, Switzerland; catherine.ngombru@rdls.nestle.com (C.N.-B.); biljana.bogicevic@rdko.nestle.com (B.B.); 2 Société des Produits Nestlé SA, Nestlé Research & Development, Nestléstrasse 3, CH-3510 Konolfingen, Switzerland; florac.debruyn@rd.nestle.com

**Keywords:** *Bifidobacterium*, phylogeny, antibiotic resistance

## Abstract

Strains of *Bifidobacterium longum* subsp. *infantis* (*B. infantis*) are amongst the first to colonize the infant gut, partly due to their capacity to metabolize complex human milk oligosaccharides (HMO), and are proposed to play a key role in the development of the infant gut. Since early life, *B. infantis* supplementation is of high interest, and detailed phylogenetic, functional and safety characterization of the selected strains should be pursued. Using a combination of long and short-read sequencing technologies, we first decipher the genetic distance between different isolates of the same *B. infantis* strain. Using the same approach, we show that several publicly available genomes recapitulate this strain-level distance as compared to two of the first strains obtained in the 1950s. Furthermore, we demonstrate that the two 1950s *B. infantis* strains display different functional and safety attributes, as ATCC 15697 is resistant to streptomycin and shows a preference towards lacto-N-tetraose LNT and sialylated HMOs, while LMG 11588 is sensitive to all tested antibiotics and shows a preference towards fucosylated HMOs. Overall, our work highlights that the current diversity observed in *B. infantis* is likely underestimated and that strain selection within this subspecies must be the subject of scientific pursuit and associated evaluation.

## 1. Introduction

*Bifidobacterium* spp. present in the human gut microbiome are of key importance for gastrointestinal health throughout the entire human lifespan [1]. This genus is one of the first to establish in the human gastrointestinal tract and is predominant in the neonate gut microbiome until the age of four months [2]. It is proposed that reduced abundance of *Bifidobacterium* spp. in the gut of infants from developed countries is associated with an increased incidence of allergic and autoimmune diseases later in life [3]. The abundance of *B. infantis* is in concordance with the general decrease of the *Bifidobacterium* genus in the developing gut microbiota from infancy to adulthood. While 70% of breastfed infants from Ghana had detectable levels of *B. infantis* in their gut, no detectable levels could be found in a comparative cohort from both New Zealand and the UK [4]. A more recent study only detected a low prevalence and abundance of *B. infantis* within the faeces of Australian, South-East Asian, and Chinese infants, which contrasted to the relatively high occurrence of *B. longum* subsp. *longum* in the same subjects [5].

Strains belonging to *B. infantis* are particularly well-adapted to metabolize the indigestible human milk oligosaccharides (HMOs), which explains at least in part their abundance in the gastrointestinal tract of breast-fed infants [6]. They are also proposed to play a key role in the maturation of the immune system and the improvement of the gut barrier function in early infancy [7]. Altogether, it was hypothesized that restoring a *B. infantis*-dominant microbiota in early infancy, through the co-administration of HMOs and selected *B. infantis* strains, is beneficial for the infant immune system maturation [8].

The oldest known strains of *B. infantis* available today were isolated in the 1950s and were initially described as *Lactobacillus bifidus* [9,10]. Taxonomy of *B. longum* subsp. *infantis* has been subjected to several changes and was eventually described and accepted to be a subspecies of the *Bifidobacterium longum* [11], for which the type strain is a strain isolated in 1963 by Reuter [12], and later deposited under ATCC 15697 [13]. The *B. longum* subsp. *infantis* strain deposited at the BCCM/LMG collection under LMG 11588 (also deposited at ATCC under ATCC 17930) is yet another strain, isolated in 1950 by Norris et al. [9], which was substantially less studied than the above mentioned type strain. A recent publication shedding light on the diversity of *B. longum* subsp. *infantis* showed that the two above-mentioned strains were closely related to several other strains isolated later. If this work suggested a close affiliation between those strains, the analysis performed by Zabel and colleagues was performed using 500 core proteins, which did not enable a strong conclusion on the relationship between the two first described strains, ATCC 15697 and ATCC 17930, and other closely related strains [14].

It has recently been reported that carbohydrate utilization patterns among different strains of *B. longum* subsp. *infantis* may vary. Strains belonging to the type strain (ATCC 15697) cluster have been described to consume different types of HMOs simultaneously and equally well with a preference for neutral and sialylated HMOs [6,15,16,17]. Opposed to that, the Bi-26 strain, clustering together with LMG 11588 (ATCC 17930), was shown to be adapted to rapidly internalize and metabolize fucosylated HMOs [14]. Fucosylated HMOs are highly prevalent in the human breast milk. 2′-fucosyllactose (2′FL) is the most abundant fucosylated HMO and represents up to 45% of the total HMO content of breast milk [15,18,19]. The work from Duar et al. suggests that metabolizing neutral and sialylated HMOs equally well presents an advantage, as they showed that the preconditioned EVC001 strain could outcompete the *B. longum* subsp. *infantis* NLS strain in vitro and in three human subjects [20]. Contrary to that, it was proposed that metabolizing fucosylated HMOs in preference could provide a competitive advantage in the gut, preventing other microorganisms from accessing this highly abundant substrate present in human breast-milk [14].

Clinical trials assessing safety (as primary or secondary outcomes) of different *B. longum* subsp. *infantis* strains, namely EVC001 [21] and M-63 [22] are available. However, to date, *B. longum* subsp. *infantis* R0033, which clusters together with the LMG 11588 (ATCC 17930) strain [14], and the DSM 33361 strain for which the genome is not publicly available, are the only strains that have been extensively evaluated by regulatory authorities, and have been communicated to the US Food and Drug Administration as generally recognized as safe (GRAS) without objection [23,24].

In this work, we aim to better understand the genetic and phenotypic diversity encompassed within *B. longum* subsp. *infantis*. Furthermore, by comparing the characteristics of ATCC 15697 and LMG 11588, as representatives of the two bigger phylogenetic clusters of *B. longum* subsp *infantis*, we not only confirm that the carbohydrate metabolism of those two groups are different, but as well highlight that their genome-based safety characteristics differ, supporting the overall need for a detailed characterization of strains intended for infant nutrition.

## 2. Materials and Methods

### 2.1. Bacterial Strains

*B. longum* subsp. *infantis* type strain isolates were obtained from three different culture collections. The type strain was obtained from the American Type Culture Collection as ATCC 15697 (ATCC, Manassas, VA, USA), from the Belgian Coordinated Collection of Microorganisms as LMG 08811 (BCCM/LMG, Ghent, Belgium) and was retrieved from the Nestlé Culture Collection (NCC) under NCC 341. *B. longum* subsp. *infantis* LMG 11588 was obtained from BCCM/LMG. Upon reactivation, strains were stored within the NCC in a freeze-dried powder form. Strains were commonly cultivated in MRSc growth medium (MRS broth supplemented 0.05% *w/v* L-Cysteine), in anaerobiosis at 37 °C for 16 h.

### 2.2. DNA Preparation and Sequencing

Strains were reactivated from the NCC freeze-dried stocks in MRS broth (Oxoid AG, Pratteln, Switzerland) containing 0.05% *w/v* L-Cysteine (Sigma-Aldrich, Darmstadt, Germany).5 mL of mid-exponentially grown culture were harvested by centrifugation (3000× *g*, 5 min) and kept frozen for later DNA preparation. DNA was prepared using the QiaAmp kit (Qiagen GmbH, Hilden, Germany) for Illumina sequencing or with MagAttract HMW DNA Kit (Qiagen GmbH, Hilden, Germany) for PacBio Sequencing.

Illumina sequencing was performed as follows. Libraries were prepared following Nextera XT protocol (Illumina Inc., San Diego, CA, USA) and cleaned using the bead-based AMPure kit (Beckman Coulter Life Sciences, Brea, CA, USA). Quality was evaluated using a LabChip GX touch capillary electrophoresis system (PerkinElmer, Waltham, MA, USA) and DNA was pooled equimolarly according to the lowest sample concentration (between 2 and 10 nM). Sequencing was performed on a HiSeq 2500 sequencer (Illumina Inc., San Diego, CA, USA), using the v4 chemistry PE125 and dual indexing with index of height bases each. Illumina raw sequencing data obtained for the different isolates of ATCC 15697 (accession numbers SRR17574374, SRR17574375, SRR17574376) and LMG 11588 (accession number SRR17574377) were deposited at the Sequence Read Archive (SRA) hosted by NCBI.

PacBio sequencing was performed according to the supplier’s recommendation. The DNA library preparation was performed following the recommended protocol from PacBio: “Preparing multiplexed microbial library using the SMRTbell Express Template Prep Kit 2.0” (Part Number 101-696-100 V.07 (July 2020)). DNA quality was checked along the library preparation using the Fragment Analyzer (Agilent Technologies, Inc., Santa Clara, CA, USA) and quantified by QuBit dsDNA protocol (Thermo Fisher Scientific AG, Basel, Switzerland). Sequencing was performed on a Sequel platform with 10 h movies on an LR SMRT cell. The loading was performed by diffusion at 8:00 p.m. with 2 h of pre-extension time. The sequencing data was further assembled using the Hierarchical Genome Assembly Process (HGAP4) de novo assembly analysis application available through the SMRT Link portal (Pacific Biosciences, Menlo Park, CA, USA). The obtained PacBio assembled genome of LMG 11588 was deposited at the Joint Genome Institute (JGI) under Project ID Ga0526375.

### 2.3. Average Nucleotide Identity Based Phylogeny

All non-sequenced genomes were retrieved from public repositories (Supplementary Table S1). Average Nucleotide Identity (ANI) was computed using the OrthoANIu software (v.1.2, https://www.ezbiocloud.net/tools/orthoaniu, accessed on 12 February 2021) to compare genome assemblies [25]. The generated matrix of pairwise genome similarities (Supplementary Table S2) was further used to build a phylogenetic tree. To assess the validity of clusters of interest, *p*-values were calculated for hierarchical clustering via multiscale bootstrap resampling (*n* = 10^4^ replications) with H_0_ (null hypothesis) = cluster is not supported by data and H_1_ (alternative hypothesis) = cluster is supported by data at significance level α = 0.05.

### 2.4. Single Nucleotide Polymorphism Comparisons

A high-quality single nucleotide polymorphism (SNP) pipeline developed by the Center for Food Safety and Applied Nutrition (CFSAN SNP Pipeline v.1.0.0/FDA) [26] was used for SNP calling [27]. Publicly available genomes were used as references to map short read sequencing data obtained from LMG 11588 or from the different ATCC 15697 isolates.

### 2.5. Genome Based Safety Analysis

Putative virulence genes of *B. longum* subsp. *infantis* LMG 11588 and ATCC 15697 were searched by BLAST analysis against the publicly available VFDB [26]. Putative antibiotic resistance genes of *B. infantis* LMG 11588 and ATCC 15697 were searched for using ResFinder [27], PointFinder [28] (both software and associated databases downloaded on from https://bitbucket.org/genomicepidemiology/, accessed on 2 December 2019) and AMRFinder (v.3.10, https://github.com/ncbi/amr/wiki, accessed on 15 March 2021) [29]. Putative enzymes resulting in the production of harmful metabolites were searched for using BLAST analysis (v.2.5.0, ftp://ftp.ncbi.nlm.nih.gov/blast/executables/blast+, accessed on 15 March 2021) against previously identified genes in the *B. longum* JDM301 strain [30]. As the *B. longum* JDM301 strain has no enzyme involved in the production of biogenic amines, protein sequences of such enzymes were searched through the MetaCyc web site (https://metacyc.org, accessed on 15 September 2021) [31]. Protein sequences from bacteria for histidine decarboxylase (EC 4.1.1.22; P28577, P00862), lysine decarboxylase (EC 4.1.1.18; B6EP92, O50657, Q9L072, P0A9H3, P52095) and ornithine decarboxylase (EC 4.1.1.17; P21169, P24169) could be retrieved and used for BLAST analysis. Only hits with percent identity > 70% and coverage > 60% were considered as significant for all readouts and reported.

### 2.6. HMO Consumption Profiles

*Bifidobacterium longum* subsp. *infantis* strains LMG 11588 and ATCC 15697 were resuscitated in a basal medium (containing yeast-derived amino acids at 3% and vitamin C) with glucose as the sole carbon source (anaerobic 37 °C for 18 h) and then transferred into the same medium, replacing glucose with a mixture of five HMOs a sole carbon source (aerobically 37 °C for 24 h). The HMOs were added at a total concentration of 2% *w/w* with ratios representing human breast milk composition, respectively: 2′-fucosyllactose (2′FL) 61%, lacto-N-tetraose (LNT) 20%, 6′-sialyllactose (6′SL) 9%, difucosyllactose (diFL) 8%, 3′-sialyllactose (3′SL) 2%. All media were autoclaved at 121 °C for 15 min. Carbohydrate solutions were filter sterilized (20 μM average pore diameter) and added aseptically after autoclavating. Medium pH was monitored in real-time as a growth measure. Initial and residual HMO concentrations were quantified by liquid chromatography with fluorescence detection after labelling with 2-aminobenzamide using the protocol described earlier [32]. All HMOs were quantified against genuine analytical standards with known purity. All carbohydrates and analytical standards were obtained from Glycom A/S (Hørsholm, Denmark).

### 2.7. Phenotypic Antibiotic Resistance Profiling by Microdilution

Phenotypic antibiotic testing of the *B. longum* subsp. *infantis* strains was performed according to the recommendations made by EFSA [33] following the official ISO 10932 method. In short, bacterial strains were propagated on MRSc agar plates and grown for 48 h at 37 °C in anaerobiosis. Colonies obtained were then resuspended (McFarland turbidity of 1) in 2 mL of physiological saline buffer. Ten (10) µL of this solution were then used to inoculate 10 mL of LSM broth (for 1000 mL: 21.06 g of Iso-SensiTest broth (Thermo Fisher Scientific AG, Basel, Switzerland); 5.2 g of MRS broth (Oxoid AG, Pratteln, Switzerland) supplemented with 0.05% of cysteine (Sigma-Aldrich, Darmstadt, Germany) having a final pH of 6.7. Hundred (100) µL of inoculated medium were then transferred in each well of precoated Sensititre^TM^ antibiotic plates (Thermo Fisher Scientific AG, Basel, Switzerland), which were finally incubated for 48 h, at 37 °C, in anaerobic condition. Minimal inhibitory concentrations (MIC) were determined by reading the turbidity in each well after 48 h of incubation and were compared to the thresholds determined by EFSA for antibiotics of human and veterinary importance.

## 3. Results

### 3.1. Phylogeny of the B. longum subsp. infantis Subspecies

**Figure 1:**
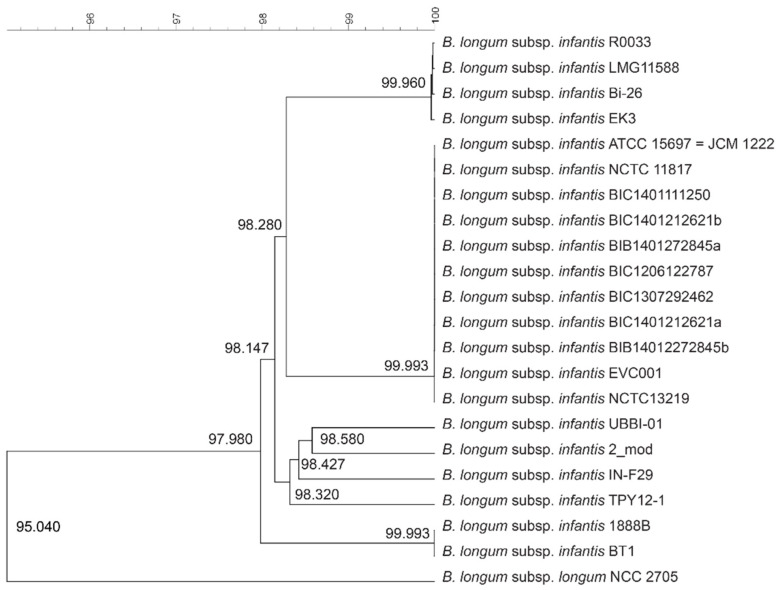
Whole genome based phylogenetic UPGMA tree of publicly available *B. longum* subsp. *infantis* strains. The genome of *B. longum* subsp. *longum* NCC 2705 was used as an outlier. Average Nucleotide Identity percentage (% ANI) is depicted at each branch node.

All publicly available genomes of *B. longum* subsp. *infantis* that had been previously compared using 500 core proteins [14] were retrieved and compared by average nucleotide identity (ANI), employing their full genomic information (Figure 1). The publicly available genome of *B. longum* subsp. *longum* NCC 2705 was added as an outlier, and clustered separately (95.040% ANI) to all *B. longum* subsp. *infantis* strains. A comparison of all *B. longum* subsp. *infantis* strains showed that several clearly distinguishable strains exist (UBBI-01, 2_mod, IN-F29, TPY12-1); all other strains being regrouped in three different clusters. A first cluster consisting of R0033, Bi-26 and EK3 share a mean homology of >99.960% ANI to the LMG11588 (ATCC 17930) strain, one of the first described isolates *of B. longum* subsp. *infantis* [9]. The second cluster, sharing 98.280% ANI with the first one, contains a set of strains, including NCTC13219 and EVC001, which were all shown to be closely related (mean homology >99.993% ANI) to the ATCC 15697 type strain, which was also one of the first described isolates of *B. longum* subsp. *infantis* [12]. These two clusters can also be differentiated from each other based on their average genome size and average GC content. Indeed, the LMG 11588 genome cluster has an average genome size of 2,599,193 bp and average GC content of 58.97%, while ATCC 15697 genome cluster has higher average genome size and average GC content (respectively 2,795,592 bp and 59.60%). Finally, the strains 1888B and BT1 were shown to be closely related to each other (>99.993% ANI) (Figure 1). These three clusters are supported by bootstrapping with *p* < 0.05 (Supplementary Figure S1).

### 3.2. SNP Variation Observed in Different Isolates of B. infantis ATCC 15697

To determine the genetic variation that can be observed within different isolates of the same strain of *B. longum* subsp. *infantis*, we sourced the ATCC 15697 type strain from different culture collections, sequenced the obtained isolates and compared them to publicly available genome sequences of this same strain using the SNP comparison pipeline developed by the US-FDA. Using this pipeline, a threshold of <20 SNPs, associated with a monophyletic phylogenetic tree topology was suggested to ensure that the pathogenic isolates are from “the same origin” [34]. Analysis showed that the number of SNPs ranges from a minimum of four up to a maximum of 15 between different isolates and sequences of the *B. infantis* type strain (Table 1).

### 3.3. Relationship of B. infantis ATCC 15697 and LMG 11588 to Other Closely Related Strains

To better understand the relationship of strains found to be closely related to ATCC 15697 and LMG 11588, the two initial strains of *B. longum* subsp. *infantis* described in the 1950s [9,12], we compared them all at SNP level, as described previously. We found that all the strains showing an ANI above 99.9% to ATCC 15697 displayed no more than 32 SNPs to that strain. Out of the 10 strains encompassed in this cluster, four strains displayed levels similar (0–16 SNPs) to what we observed previously for different isolates of the same strain. The other six strains displayed a slightly higher number of polymorphisms (26–32 SNPs) (Table 2). Similarly, all strains showing an ANI of more than 99.9% to LMG 11588 displayed not more than 13 SNPs to that strain (Table 3). 

A total of 231 “raw” SNPs were identified when mapping the LMG 11588 reads against the EK3 strain genome assembly. When examining all SNPs, it was found that a lot of clustered SNPs were identified in regions of the EK3 strain genome assembly where stretches of undetermined nucleotides (“N”) were present. These regions were considered as unreliable, and associated SNPs were removed, resulting in a final count of 13 SNPs difference between the EK3 and LMG 11588 strains.

### 3.4. HMO Consumption Profiles of B. infantis ATCC 15697 and LMG 11588

**Figure 2:**
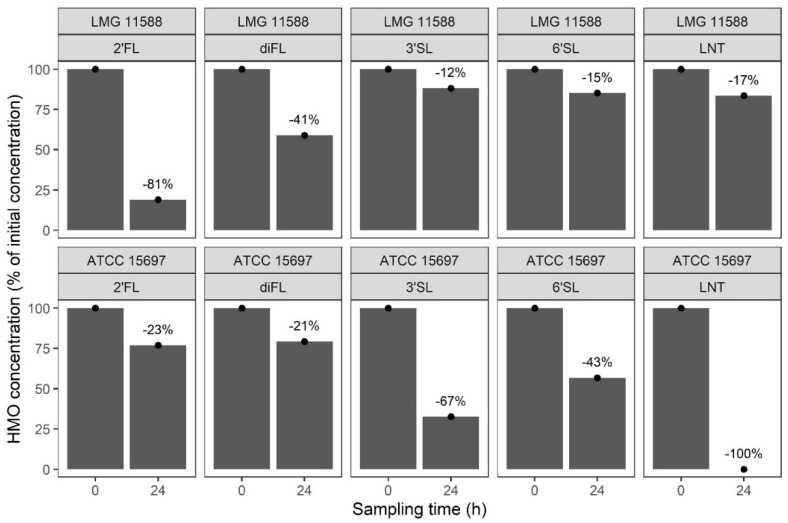
HMO consumption profile of *B. infantis* ATCC 15697 and LMG 11588 grown for 24 h in a medium containing a mix of five HMOs as sole carbon source.

It was previously shown that two HMO consumption patterns exist among strains of *B. longum* subsp. *infantis* [14,15,16,17]. To determine if the two strains isolated in the 1950s (ATCC 15697 and LMG 11588) reflect those differences, both strains were cultured for 24 h in a medium containing a mix of five HMOs as the sole carbon source. Consumption of each individual HMO was measured in the culture supernatant using liquid chromatography with fluorescence detection. Results showed that the type strain ATCC 15697 consumed the entire LNT pool present in the mix (−100%), had a preference towards sialylated HMOs (3′SL −67%; 6′SL −43%), and consumed the fucosylated HMOs to a lower degree (2′FL, −23%; diFL, −21%) (Figure 2). The observed consumption profile of the ATCC 15697 strain reflects previously described ones for this strain and other closely related strains (e.g., EVC001) [20].

In contrast, the LMG 11588 strain preferably consumed both fucosylated HMOs present in the mix (2′FL, −81%; diFL, −41%) and metabolized other HMOs present to different degrees (LNT, −17%; 3′SL, −12%; 6′SL, −15%) (Figure 2). This efficient consumption of fucosylated HMOs has already been described previously for another closely related strain (Bi-26) [14].

### 3.5. Phenotypic Antibiotic Resistance Profiles and rpSL Gene Mutation in B. infantis ATCC 15697 and LMG 11588

Antibiotic resistance was assessed using the microdilution method. Obtained minimal inhibitory concentrations (MICs) were compared to the cut-off determined by EFSA. Analysis showed that *B. infantis* LMG 11588 was sensitive to all antibiotics tested, while the type strain *B. infantis* ATCC 15697 was resistant to streptomycin since the MIC obtained for this antibiotic was of 512 µg/mL and higher than the resistance cut-off determined by EFSA (Table 4).

The observed streptomycin resistance in the ATCC 15697 strain could be explained by the presence of a point mutation in the *rpSL* gene of this strain (A to G replacement occurring at nucleotide 128) (Table 5). This mutation was initially described in *B. breve* [35] and was also detected in several *Bifidobacterium* strains, including *B. longum* subsp. *infantis* M-63 [36]. While screening the LMG 11588 strain genome, the *mer(A)* gene was identified. This gene was not found in the ATCC 15697 genome. Mercury resistance induced by *mer* genes was initially suspected to be positively associated with multidrug resistance in Gram negative human fecal bacteria [37]. However, this association could not be shown for Gram positive bacteria [38].

### 3.6. Genetic Organization around Mutated rpSL Gene in B. infantis ATCC 15697 and LMG 11588

**Figure 3:**
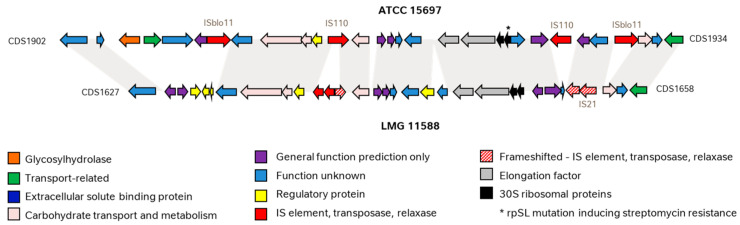
Comparison of the genomic organization surrounding the rpSL gene of *B. longum* subsp. *infantis* ATCC 15697 and LMG 11588. Homologous DNA portions are indicated in grey. Functional classes are represented by different colors.

The genome of ATCC 15697 revealed that the mutated *rpSL* gene (Blon_1925) was encompassed by different transposases. Two IS110 family proteins were found in the close vicinity (Blon_1914 and Blon_1928) which shared a relatively low level of identity to each other (48%). Two ISBlo11 family transposases (Blon_1909 and Blon_1931) with an identity of 100% encompassing the full genomic region were found further away. Even if the two ISBlo11 transposases are oriented in the same direction, they contain inverted repeats typical of this transposase family, suggesting that they are fully functional. Supporting this, ISblo11 transposases were previously found to be fully functional, and were even proposed to be employed for a transposon-mediated mutagenesis system in bifidobacteria [39]. The organization of genes surrounding the *rpSL* gene of LMG 11588 (KY279_001650) was found to be slightly different. An IS21 type transposase and its IS21-like element helper ATPase (KY279_001655 and KY279_001654 respectively) are located downstream of the *rpSL* gene, but were found to be frameshifted. Upstream of the *rpSL* gene of LMG 11588, a frameshifted transposase (KY279_001639), an integrase from the IS3 family (KY279_001638), and a relaxase (KY279_001637) were also found (Figure 3).

### 3.7. Presence of Other Safety Related Genes

We screened both LMG 11588 and ATCC 15697 genomes for the presence of virulence encoding genes as well as genes encoding enzymes responsible for the production of potentially harmful metabolites. No genes related to the production of virulence factors could be detected in both genomes. Genes encoding conjugated bile salt hydrolase (CBSH), D-lactate dehydrogenase (DLD) and nitroreductase (ND) could be observed in both genomes. Beta-glucuronidase genes could be detected independently in the JDM301 (BLJ_0623) and LMG 11588 (KY279_001620), but not in ATCC 15697 genome (Table 6). No genes known to code for tyrosine decarboxylase, ornithine decarboxylase, histidine decarboxylase, lysine decarboxylase, arylsulfatase or azoreductase could be found in any of the LMG 11588 and ATCC 15697 genomes.

## 4. Conclusions and Discussion

According to our whole genome comparative analysis, a significant number of *B. infantis* strains described today are falling into two distinct clusters, which are grouped around two isolates obtained in the 1950s: ATCC 15697 and LMG 11588 (ATCC 17930). All strains belonging to the same cluster are sharing an average nucleotide identity (ANI) of more than 99.9% with one of those two strains, and most of them displayed a low number of SNPs (<21) compared to one of the two isolates from the 1950s. The SNP analysis pipeline used in this work has been developed by UCFSAN-FDA to determine and track the source of different foodborne contaminating isolates. Based on several outbreak investigations, it was considered that isolates sharing fewer than 21 SNPs over their whole genome can be considered as originating from the same source [34]. Our analysis revealed a maximum of 15 SNPs in several isolates of the same ATCC 15697 strain, representing the expected genetic variation displayed in a single strain of *B. longum* subsp. *infantis*. These differences may be caused by the strain propagation or simply be the result of different sequencing and assembly procedures.

Our results demonstrate that the two oldest known isolates of *B. longum* subsp. *infantis* (ATCC 15697 and LMG 11588) are closely related to several of the strains that have been isolated and sequenced in the last decades, suggesting that the strains organized in the two corresponding clusters are highly clonal. Those results partially repeat a situation already observed within different strains of *B. animalis* subsp. *lactis* [40]. Even if several publicly available genomes share strain-level genetic distance to the two strains initially isolated by Norris et al. [9] and Reuter et al. [12], it is worth noting that, through evolution, several of those strains might have diverged phenotypically, as a few distinct SNPs in coding or regulatory regions might create phenotypic differentiation [41]. Further research is therefore required to understand the potential implications of SNP level differences within the two aforementioned clusters.

An important feature studied in *B. infantis* strains is their adaption to use HMOs in a very particular way. Until recently, the HMO consumption patterns displayed by *B. infantis* strains were believed to be relatively homogenous, consuming neutral and sialylated HMOs simultaneously and equally well [6,15,16,17,20]. Recently, Zabel and colleagues demonstrated that members of this subspecies exhibit distinct metabolic profiles, showing that the Bi-26 strain had a strong preference for fucosylated HMOs [14], suggesting the existence of a wider diversity in the *B. infantis* subspecies.

Our results on HMO consumption patterns of both ATCC 15697 and LMG 11588 are consistent with previous observations made for some strains belonging to the same cluster. We demonstrated that when grown on a mix of several 5 HMOs (2′FL, diFL, LNT, 3′SL and 6′SL), ATCC 15697 consumes LNT particularly well, but also consumes other sialylated HMO, confirming findings obtained previously on the same strain [6,15,16,17], and a closely related strain, namely EVC001 (>99.9% ANI) [20]. Similarly, the obtained HMO consumption profile of LMG 11588, showing a preference towards fucosylated HMOs, has also been observed for the closely related Bi-26 strain [14]. As can be expected from their close genetic relationship (>99.9% ANI), LMG 11588 and Bi-26 have the exact same HMO utilization gene setups. It is therefore interesting that despite the fact that LMG 11588 displays a non-functional ABC transporter system predicted to import LNT (Blon_2175 to Blon_2177 in ATCC 15697) [14], it is still able to partly metabolize LNT, suggesting the existence of a potential secondary LNT transport mechanism in that strain, which is today still to be described. On the other hand, Zabel and colleagues have demonstrated in Bi-26 that expression of different fucose utilization genes were upregulated in the presence of 2′FL, 3′FL or diFL, which could partly explain the efficient utilization of those substrates by the close relative LMG 11588 [14].

The antibiotic resistance profile of strains that are intentionally added to food or feed is today particularly scrutinized by regulatory authorities [33,42].The increase in antibiotic resistance around the globe represents a serious public health challenge. Potentially transferable antibiotic resistances should particularly be avoided, as it has been hypothesized that this could represent a reservoir of resistance and could be transferred to other bacteria present in the gastro-intestinal tract [43]. The two above-described *B. longum* subsp. *infantis* strains also differ in this respect. Our phenotype analysis exposed streptomycin resistance in *B. infantis* ATCC 15697 as described by Kim and colleagues [44], but not in LMG 11588. *B. infantis* ATCC 15697, and closely related strains harbor a mutation in the 30S ribosomal gene *rpSL* that was previously shown to encode a streptomycin resistance in *B. breve* [35] and was shown to be present in other bifidobacterial strains, including *B. longum* subsp. *infantis* M-63 [36]. In the presence of streptomycin, the *rpSL* mutation conferring resistance occurred at a relatively high frequency (10^−8^ to 10^−9^) [35]. Our analysis of the region surrounding the mutated rpSL gene in ATCC 15697 showed the presence of transposases, of which at least two are likely functional. Similar IS elements (ISBlo11) have been shown to be functional and even proposed to be employed for the development of a transposon-mediated mutagenesis system in bifidobacteria [39], suggesting that this region in ATCC 15697 and other *B. infantis* strains from the same cluster might be transferable to other microorganisms. On the other side, the gene setup surrounding non-mutated *rpSL* genes of *B. infantis* LMG 11588 should not be of concern. First, the *rpSL* gene does not harbor the mutation encoding streptomycin resistance. Second, the transposases surrounding it should not enable transfer of the region as they are from different families (IS3 and IS21) and partly non-functional (frameshifted). Today, streptomycin is still an actively used antibiotic [45]. Hence, further studies are required to assess the transferability of this genetic region in *B. infantis* ATCC 15697 and closely related strains and clarify the potential safety issue this might represent.

Overall, our results highlight that the current diversity observed in *B. infantis* is likely underestimated. Indeed, the genetic diversity observed in a number of publicly available strains suggest that they are clonally related. We further showed that all the closely related strains described in this work are regrouped in two distinct clusters, each of them containing one of the two first isolated *B. infantis* strains obtained in the 1950s by Norris et al. [9] and Reuter et al. [12], ATCC 15697 and LMG 11588, respectively. Finally, we can confirm that those two representative strains harbor different functional and safety attributes, highlighting the need for a detailed strain evaluation when developing commercial *B. infantis* products intended for early life supplementation.

## Figures and Tables

**Table 1:** Genomic SNP level comparison of different isolates of the *B. infantis* ATCC 15697 (type strain) sourced from different culture collection. All three isolates were compared to the two publicly available genomes of the strain. Genome accession numbers and sizes are indicated in parentheses.

Public Genome Version	LMG 08811	ATCC 15697	NCC 341
ATCC 15697 (NC_017219.1; 2,828,958 bp)	5	4	13
ATCC 15697 (NC_011593.1; 2,832,748 bp)	8	14	15

**Table 2:** Number of SNPs found between the *B. infantis* ATCC 15697 sequenced strain and closely related publicly available genomes (ANI > 99.9%).

Strain Name (Genome Size in Base Pair)	ATCC 15697
NCTC13219 (2,602,591 bp)	0
NCTC11817 (2,832,748 bp)	5
BIB1401272845a (2,791,524 bp)	13
BIC1401212621a (2,791,569 bp)	31
BIC1401212621b (2,821,883 bp)	16
BIB1401272845b (2,786,838 bp)	30
Strain_6 (EVC001) (2,832,850 bp)	28
BIC1206122787 (2,789,037 bp)	26
BIC1401111250 (2,793,888 bp)	33
BIC1307292462 (2,879,623 bp)	32

**Table 3:** Number of SNPs found between the *B. infantis* LMG 11588 sequenced strain and closely related publicly available genomes (ANI > 99.9%).

Strain Name (Genome Size in Base Pair)	LMG 11588
R0033 (2,615,717 bp)	16
Bi-26 (2,569,437 bp)	4
Ek3 (2,564,809 bp)	13 (231 ^1^)

^1^ SNPs identified in genomic regions where stretches of undetermined nucleotides (“N”) were present.

**Table 4:** Phenotypic resistance of *B. infantis* ATCC 15697 and LMG 11588 to relevant antibiotics as determined by EFSA. Minimal inhibitory concentrations (MICs) were determined using the microdilution method and are expressed in µg/mL.

Antibiotic	ATCC 15697	LMG 11588	Cut-Off Determined by EFSA
Gentamicin (µg/mL)	1.0	32.0	64.00
Streptomycin (µg/mL)	512.0	16.0	128.00
Tetracyclin (µg/mL)	2.0	2.0	8.00
Erythromycin (µg/mL)	0.125	0.5	1.00
Clindamycin (µg/mL)	0.125	0.125	1.00
Chloramphenicol (µg/mL)	1.0	2.0	4.00
Ampicillin (µg/mL)	0.13	0.25	2.00
Vancomycin (µg/mL)	1.0	0.5	2.00

**Table 5:** rpSL gene sequence at position 128 (of *B. breve* rpSL gene).

Strain	Streptomycin Resistance	rpSL Gene, Positions 122–134
*B. breve* strain Yakult [35]	resistant	ccccga**G**gaagcc
*B. infantis* ATCC 15697	resistant	ccccga**G**gaagcc
*B. infantis* LMG 11588	sensitive	ccccga**A**gaagcc

**Table 6:** Significant BlastP hits for putative enzymes implicated in the production of harmful metabolites in B.infantis ATCC 15697 and LMG 11588 genomes.

Enzymes	JDM301 Locus_Tag	LMG 11588 Locus_Tag (% Identity; Coverage)	ATCC 15697 Locus_Tag (% Identity; Coverage)
Conjugated bile salt hydrolase (CBSH)	BLJ_0948	KY279_001316 (98.74; 0.99)	Blon_1453 (98.42; 1)
D-lactate dehydrogenase (DLD)	BLJ_1306	KY279_000900 (98.50; 1)	Blon_0845 (98.50; 1)
	BLJ_1436	KY279_000777 (96.95; 1)	Blon_0718 (97.26; 1)
Nitroreductase (NR)	BLJ_1980	KY279_002229 (99.21; 1)	Blon_2447 (99.61; 1)

## Data Availability

All assembled *B. longum* subsp. *infantis* genomes used in this study are listed in Supplementary Table S1. Raw sequencing data used for the reported SNP analysis have been deposited at the Sequence Read Archive (SRA) hosted by NCBI (accession numbers SRR17574374, SRR17574375, SRR17574376 and SRR17574377). The annotated PacBio LMG 11588 genome is deposited at the JGI under Project ID Ga0526375.

## Supplementary Materials

The following supporting information can be downloaded at: https://www.mdpi.com/article/10.3390/microorganisms10020203/s1, Figure S1: Average nucleotide difference with bootstrapping *p*-values for clusters of interest, Table S1: List of *B. longum* subsp. *infantis* strains and genomes used in this study, Table S2: Average Nucleotide Identity (% ANI) matrix of compared genomes.
