# Correction: Duboux et al. Phylogenetic, Functional and Safety Features of 1950s *B. infantis* Strains. *Microorganisms* 2022, *10*, 203

**DOI:** 10.3390/microorganisms13122665

**Published:** 2025-11-24

**Authors:** Stéphane Duboux, Catherine Ngom-Bru, Florac De Bruyn, Biljana Bogicevic

**Affiliations:** 1 Société des Produits Nestlé SA, Nestlé Research, Route du Jorat 57, CH-1000 Lausanne 26, Switzerland; catherine.ngombru@rdls.nestle.com (C.N.-B.); biljana.bogicevic@rdko.nestle.com (B.B.); 2 Société des Produits Nestlé SA, Nestlé Research & Development, Nestléstrasse 3, CH-3510 Konolfingen, Switzerland; florac.debruyn@rd.nestle.com

In the original publication [1], two references Reuter G., 1963 and Reuter G., 1971 were not cited. Both citations have now been inserted as [12] and [13] in the third paragraph of part of “Introduction”. With this correction, the order of some references has been adjusted accordingly. The detailed information of the two newly added references is presented below:

12. Reuter, G. Comparative studies on the bifidus flora in the feces of infants and adults. with a contribution to classification and nomenclature of bifidus strains. *Zentralbl. Bakteriol. Orig.* **1963**, *191*, 486–507.
13. Reuter, G. Designation of type strains for *Bifidobacterium* species. *Int. J. Syst. Bacteriol.* **1971**, *21*, 273–275.

In the Introduction section, the third paragraph should read the following:

“The oldest known strains of *B. infantis* available today were isolated in the 1950s and were initially described as *Lactobacillus bifidus* [9,10]. Taxonomy of *B. longum* subsp. *infantis* has been subjected to several changes and was eventually described and accepted to be a subspecies of the *Bifidobacterium longum* [11], for which the type strain is a strain isolated in 1963 by Reuter [12], and later deposited under ATCC 15697 [13]. The *B. longum* subsp. *infantis* strain deposited at the BCCM/LMG collection under LMG 11588 (also deposited at ATCC under ATCC 17930) is yet another strain, isolated in 1950 by Norris et al. [9], which was substantially less studied than the above mentioned type strain. A recent publication shedding light on the diversity of *B. longum* subsp. *infantis* showed that the two above-mentioned strains were closely related to several other strains isolated later. If this work suggested a close affiliation between those strains, the analysis performed by Zabel and colleagues was performed using 500 core proteins, which did not enable a strong conclusion on the relationship between the two first described strains, ATCC 15697 and ATCC 17930, and other closely related strains [14].”

In Sections 3.1 and 3.3, reference [12] was added.

The addition of the above citations is also reflected in the Conclusions and Discussion section, second paragraph and sixth paragraph:

Second paragraph: “Our results demonstrate that the two oldest known isolates of *B. longum* subsp. *infantis* (ATCC 15697 and LMG 11588) are closely related to several of the strains that have been isolated and sequenced in the last decades, suggesting that the strains organized in the two corresponding clusters are highly clonal. Those results partially repeat a situation already observed within different strains of *B. animalis* subsp. *lactis* [40]. Even if several publicly available genomes share strain-level genetic distance to the two strains initially isolated by Norris et al. [9] and Reuter et al. [12], it is worth noting that, through evolution, several of those strains might have diverged phenotypically, as a few distinct SNPs in coding or regulatory regions might create phenotypic differentiation [41]. Further research is therefore required to understand the potential implications of SNP level differences within the two aforementioned clusters.”

Sixth paragraph: “Overall, our results highlight that the current diversity observed in *B. infantis* is likely underestimated. Indeed, the genetic diversity observed in a number of publicly available strains suggest that they are clonally related. We further showed that all the closely related strains described in this work are regrouped in two distinct clusters, each of them containing one of the two first isolated *B. infantis* strains obtained in the 1950s by Norris et al. [9] and Reuter et al. [12], ATCC 15697 and LMG 11588, respectively. Finally, we can confirm that those two representative strains harbor different functional and safety attributes, highlighting the need for a detailed strain evaluation when developing commercial *B. infantis* products intended for early life supplementation.”

The authors state that the scientific conclusions are unaffected. This correction was approved by the Academic Editor. The original publication has also been updated.
