# Supplementary figures and images for: Phylogenetic, Functional and Safety Features of 1950s B. infantis Strains
Source: Microorganisms. 2022 Jan 18;10(2):203. doi: 10.3390/microorganisms10020203 (PMC8879182; doi:10.3390/microorganisms10020203)

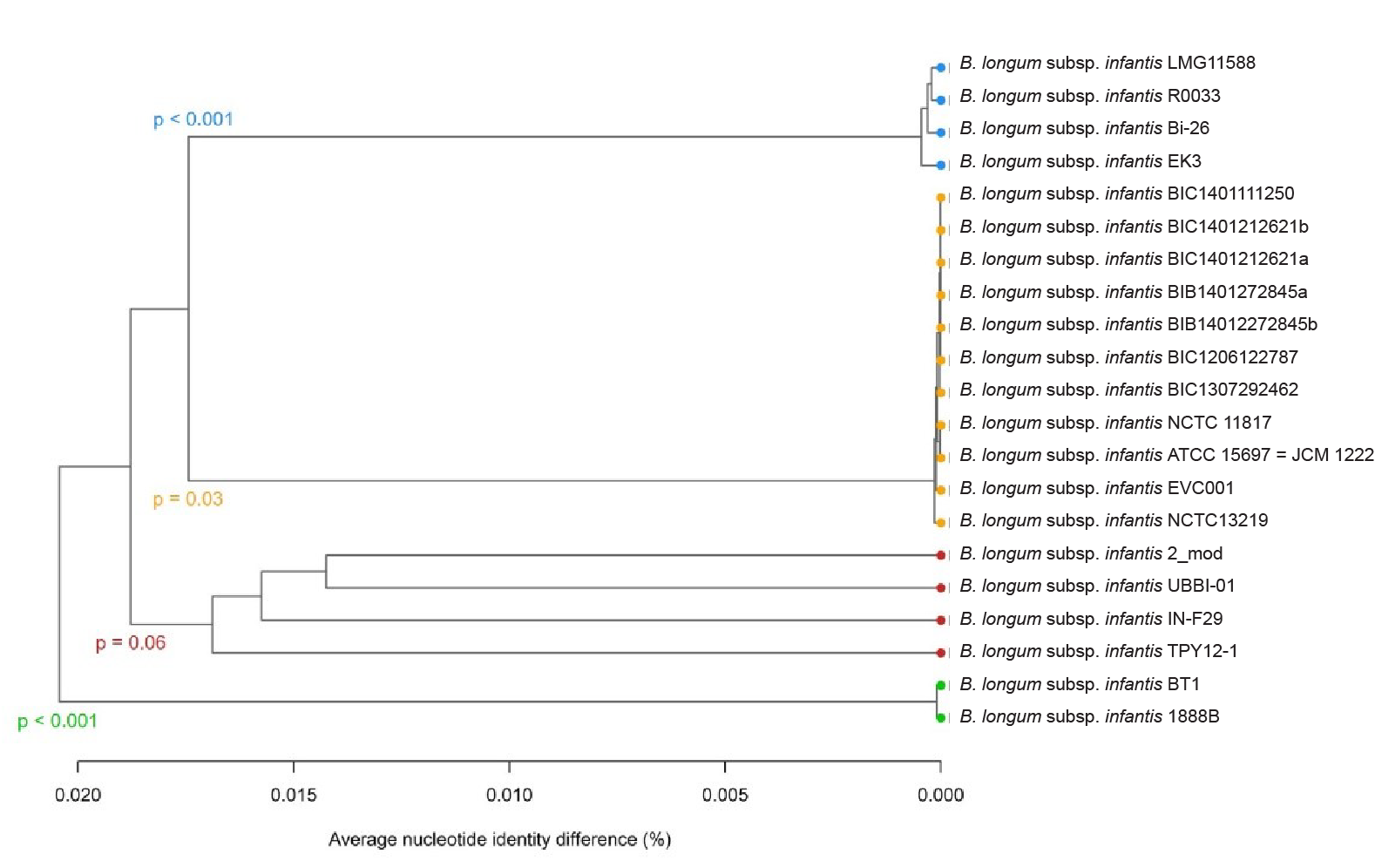

Supplement: Supplementary file 1 [file microorganisms-10-00203-s001.zip › Figure S1 rev.png]
